# Thymoquinone inhibited vasculogenic capacity and promoted mesenchymal-epithelial transition of human breast cancer stem cells

**DOI:** 10.1186/s12906-021-03246-w

**Published:** 2021-03-04

**Authors:** Sanya Haiaty, Mohammad-Reza Rashidi, Maryam Akbarzadeh, Ahad Bazmany, Mostafa Mostafazadeh, Saba Nikanfar, Zohre Zibaei, Reza Rahbarghazi, Mohammad Nouri

**Affiliations:** 1 Department of Biochemistry and Clinical Laboratories, Tabriz University of Medical Sciences, Tabriz, Iran; 2 Student Research Committee, Tabriz University of Medical Sciences, Tabriz, Iran; 3 Stem Cell and Regenerative Medicine Institute, Tabriz University of Medical Sciences, Tabriz, Iran; 4 Department of Biochemistry, Erasmus University Medical Center, Rotterdam, the Netherlands; 5 Department of Pathobiology, Faculty of Veterinary Medicine, Ferdowsi University Of Mashhad, Mashhad, Iran; 6 Research Center of Infectious Diseases and Tropical Medicine, Tabriz University of Medical Science, Tabriz, Iran; 7 Stem Cell Research Center, Tabriz University of Medical Sciences, Imam Reza St., Golgasht St, Tabriz, Iran; 8 Departmnt of Applied Cell Sciences, Faculty of Advanced Medical Sciences, Tabriz University of Medical Sciences, Tabriz, Iran

**Keywords:** Thymoquinone, Vasculogenic mimicry, Breast Cancer stem cells, Wnt3a/PI3K signaling pathways

## Abstract

**Background:**

Vasculogenic mimicry (VM) is characterized by the formation of tubular structure inside the tumor stroma. It has been shown that a small fraction of cancer cells, namely cancer stem cells (CSCs), could stimulate the development of vascular units in the tumor niche, leading to enhanced metastasis to the remote sites. This study aimed to study the inhibitory effect of phytocompound, Thymoquinone (TQ), on human breast MDA-MB-231 cell line via monitoring Wnt/PI3K signaling pathway.

**Methods:**

MDA-MB-231 CSCs were incubated with different concentrations of TQ for 48 h. The viability of CSCs was determined using the MTT assay. The combination of TQ and PI3K and Wnt3a inhibitors was examined in CSCs. By using the Matrigel assay, we measured the tubulogenesis capacity. The percent of CD24^−^ CSCs and Rhodamine 123 efflux capacity was studied using flow cytometry analysis. Protein levels of Akt, p-Akt, Wnt3a, vascular endothelial-cadherin (VE-cadherin), and matrix metalloproteinases-2 and -9 (MMP-2 and -9) were detected by western blotting.

**Results:**

TQ decreased the viability of CSCs in a dose-dependent manner. The combination of TQ with PI3K and Wnt3a inhibitors reduced significantly the survival rate compared to the control group (*p* < 0.05). TQ could blunt the stimulatory effect of vascular endothelial growth factor (VEGF), epidermal growth factor (EGF), fibroblast growth factor (FGF) on CSCs (*p* < 0.05). The vasculogenic capacity of CSCs was reduced after being-exposed to TQ (*p* < 0.05). Western blotting revealed the decrease of CSCs metastasis by suppressing MMP-2 and -9. The protein level of VE-cadherin was also diminished in TQ-treated CSCs as compared to the control cell (*p* < 0.05), indicating inhibition of mesenchymal-endothelial transition (MendT). TQ could suppress Wnt3a and PI3K, which coincided with the reduction of the p-Akt/Akt ratio. TQ had the potential to decrease the number of CD24^−^ CSCs and Rhodamine 123 efflux capacity after 48 h.

**Conclusion:**

TQ could alter the vasculogenic capacity and mesenchymal-epithelial transition of human breast CSCs in vitro. Thus TQ together with anti-angiogenic therapies may be a novel therapeutic agent in the suppression of VM in breast cancer.

## Background

Breast cancer is touted as the most common mammary gland malignancy in females globally [1]. Numerous attempts have been made to decrease the breast cancer mortality rate in patients with breast cancer but it is still the main cause of mortality in developing and industrialized countries [2]. In solid tumors, the diffusion of oxygen and nutrients is critical to promote the expansion and proliferation of cancer cells [3]. In this regard, the development of de novo blood vessels, so-called angiogenesis, is an efficient way to supply tumor cells’ demands [4]. Besides, the expansion of the vascular bed into the tumor mass could facilitate the invasion and metastasis of the remote sites [5]. Angiogenesis is defined as the formation of new blood vessels from the pre-existing vascular network [6–8]. Besides the angiogenesis, a complementary angiogenic mechanism named vasculogenesis and/or VM could promote the formation of vascular structure inside the tumor stroma [9, 10]. In the process of vasculogenesis different progenitor cells could be recruited to the site of injury and trans-differentiate into mature endothelial cells (ECs) to increase blood supply to the hypoxic cells [11]. Previous studies have confirmed the existence of vasculogenesis in multiple malignancies, such as ductal breast carcinoma, melanoma, etc. [5].

In most circumstances, the conventional therapeutic protocol could not eradicate the cancer cells because of resistance to chemotherapies [12]. It has been shown that a small fraction of cancer cells namely CSCs used different strategies to circumvent the therapeutic approaches and immune cell responses [13]. For example, CSCs have the potential to trans-differentiate into different lineages such as ECs which is termed also MendT. These capacities help CSCs to promote microvascular density in the target cancerous niche [14]. According to previously published data, cells with identical CD24^−^/CD44^+^ markers possess typical CSCs during the progression of breast cancers, leading to poor prognosis [15]. As above-mentioned, breast CSCs like other CSC types could stimulate vascular formation via VM activity [5]. Multiple signaling pathways and effectors such as PI3K, Wnt cascades are closely associated with the VM properties [5, 16, 17]. For instance, it has been shown that PI3K increases VM capacity via the up-regulation of different genes like MT1-MMP and MMP-2, leading to enhanced vasculogenic outcomes [14]. Therefore, the control of angiogenesis signaling pathways in CSCs or cancer ECs could be an efficient approach to inhibit tumor expansion and metastasis [18].

TQ is a natural phyto-compound driven from *Nigella sativa Linn* has been used in traditional medicine for a variety of complaints in countries from the Mediterranean region to Western Asia [19]. Several studies have indicated that the seeds and oil of TQ have significant anti-parasitic, anti-microbial, anti-oxidant, anti-inflammatory, and immuno-modulatory properties with very low toxicity [20]. Additionally, in vitro and in vivo systems showed the potency of TQ to exert an anticancer activity against various tumor cells via engaging different mechanisms such as anti-angiogenic capacity [19, 21].

The exact mechanism of TQ participated in the suppression of breast cancer is the subject of interest. This study aimed to assess the anti-angiogenic capacity of TQ in the human breast MDA-MB-231 cell line. The possible effect of TQ on stemness features and MendT rate were also measured by monitoring the activity of PI3K/Akt and Wnt/β-catenin signaling pathways. We hope that the results of this experiment could help us to understand the underlying mechanisms participating in the tumoricidal activity of the TQ.

## Methods

### Cell culture protocol and expansion

In the current experiment, we used a CSC-like phenotype namely the MDA-MB-231 cell line (NCBI code: C10684). Cells were purchased from Iranian Cell Bank (Pasture Institute, Tehran) and expanded in RPMI 1640 (Gibco, USA) culture medium. The medium was enriched with 10% FBS (Gibco) and 1% Pen-Strep (Gibco). Culture flasks containing MDA-MB-231 were maintained at 37 °C in a humidified atmosphere of 95% air with 5% CO_2_. To passage the cells, we used an enzymatic solution of 0.25% Trypsin-EDTA (Gibco, USA). Cells at passages 3–6 were subjected to the subsequent experiments.

### MTT survival assay

#### Treatment of MDA-MB-231 cells with TQ

An initial number of 1 × 10^4^ MDA-MB-231 cells were plated in each well of 96-well plates (SPL, Korea) and incubated under the conventional condition and allowed the cells to reach 70–80% confluence. CSCs were treated with different concentrations of TQ (Cat no: 15039; Cayman; USA) (0.781, 1.562, 3.125. 6.25, 12.5, 25, 50, 100, 200 and 400 μM) in the culture medium supplemented with 1% FBS (fetal bovine serum). After completion of the incubation period, the supernatants were discarded and replaced with 30 μl of 5 mg/ml MTT solution (Sigma-Aldrich). The plates were maintained at 37 °C for 4 h followed by the addition of the DMSO solution to dissolve the insoluble formazan crystals. The plates were shaken gently for 15 min. Finally, the optical density of each well was measured at 630 nm using a microplate reader (Hiperion MPR4^+^, Germany). Three sets of MTT assays were conducted. According to previous data, we selected 10 and 30 μM TQ for different analyses [22, 23].

#### Measuring MDA-MB-231 cells viability using Wnt3a and PI3K inhibitors

To this end, MDA-MB-231 cells were cultured in 96-well plates as above-mentioned and pre-treated with Wnt3a (LGK974, Cat no: 14072; Cayman; USA) and PI3K (Ly294002, Cas no: 934389–88-5; Sigma-Aldrich) inhibitors 24 h before exposure to 10 and 30 μM TQ [24, 25]. Thereafter, cells were kept at 37 °C for 48 h. Finally, the survival rate was calculated using the MTT assay. This assay was performed in triplicate. Cell viability was expressed as % of non-treated control cells.

#### Measuring MDA-MB-231 cells viability after treatment with TQ and VEGF, EGF, and FGF

Cells were pre-treated with 10, 30 μM TQ for 48 h followed by incubation with 10 ng/ml VEGF (Cat no: C64423; Promocell), FGF (Cat no: F0291; Sigma-Aldrich), and EGF (Cat no: SRP3027; Sigma-Aldrich) for next 24 h. Finally, the viability of each group was calculated using MTT and expressed as % of the non-treated control MDA-MB-231 cells.

### Tubulogenesis assay

50 μl pre-cooled growth factor-reduced Matrigel (Cat no: 356230; Corning) was transferred onto each well of a 96-well plate and allowed to solidify at 37 °C. Then, a 200 μl culture medium containing 1% FBS and 2.5 × 10^4^ cells pre-treated with TQ was overlaid [26]. The plates were maintained inside the culture incubator and the formation of tubes monitored during the first 24 h. In the current experiment, the tube area, perimeter, and the number was calculated in five random fields using ImageJ software (Version 1.52a). The experiment was performed in triplicate.

### Flow cytometric analysis of CD24 in MDA-MB-231 cells after exposure to TQ

TQ-treated cells were collected using a 0.25% Trypsin-EDTA solution and washed twice with PBS (Phosphate-buffered saline) Then, cells were incubated in 100 μl PBS with 1 μg/ml of FITC-conjugated CD24 antibody (Order no: 130–095-952; Miltenyi Biotec; Germany) according to the manufacturer’s instructions. After twice PBS wash, the percent of CD24^+^ cells were calculated using BD FACSCalibur and FlowJo software (version 7.6.1).

### Flow cytometric analysis of Rhodamine123 efflux capacity

MDA-MB-231 cells (3 × 10^5^ cells/well) were seeded in 6-well plates in a culture medium containing 1% FBS and 10 and 30 μM. After 48 h, 1 mg/ml Rhodamine 123 (Cas no: 62669–70-9; Sigma-Aldrich) at 37 °C for 40 min followed by twice PBS washes. Thereafter, cells were collected and resuspended in 500 μl PBS and subjected to the flow cytometry system (BD Bioscience, USA). Data were analyzed using FlowJo software.

### Spheroid formation assay

MDA-MB-231 cells harvested and single-cell suspensions at a density of 1 × 10^4^ cells per 20 μl of RPMI culture medium containing 1% FBS, 0.1% gelatin carefully placed under the lids of cell culture plate and were inverted over the culture plates. Then the plates were incubated at 37 °C under a humidified condition with 5% CO_2_. After 3 days of incubation, the spheroids formation was monitored by using the inverted microscope (Labomed, USA).

### Western blotting

Both control and treated MDA-MB-231 cells were collected and total protein content was harvested using a protein extraction buffer containing Triton X100, NP40, Tris-HCl, EDTA, NaCl, Sodium Deoxycholate, SDS (sodium dodecyl sulfate), enriched with protease inhibitor cocktail. Lysates were then centrifuged at 12000 ***g*** for 20 min at 4 °C. Then, the supernatant removed and the concentration of total cellular protein was determined using the Bradford assay. The equal amount of extracted protein was separated by using 10% SDS-PAGE and transferred to the PVDF membrane. After that, the membranes were blocked with 2% skim milk at RT for 1 h and then incubated with primary antibodies: β-actin (1:300; sc-47,778), VE-cadherin (1:200; sc-52,751), Akt (1:1000; E-AB-30471,), p-Akt (1:100; sc-271,966,), MMP2 (1:200; sc-10,736,), MMP9 (1:100; sc-393,859), Wnt-3a (1:100; sc-74,537) at 4 °C overnight. The next day, membranes were incubated with appropriate HRP-secondary antibodies (1:1000; anti-rabbit sc-2357). The immunoblots were visualized using ECL reagent and X-ray films. The density of each band was determined using ImageJ software.

### Statistical analysis

Data are expressed as mean ± SD. To find the statistical significance, we performed One-Way ANOVA with Tukey post hoc analysis. *p* < 0.05 value was considered statistically significant. In the brackets, the significant differences were shown using asterisks as follows; *p < 0.05; **p < 0.01; ***p < 0.001 and ****p < 0.0001.

## Results

### TQ diminished viability of MDA-MB-231 cells in a dose-dependent manner

**Fig. 1:**
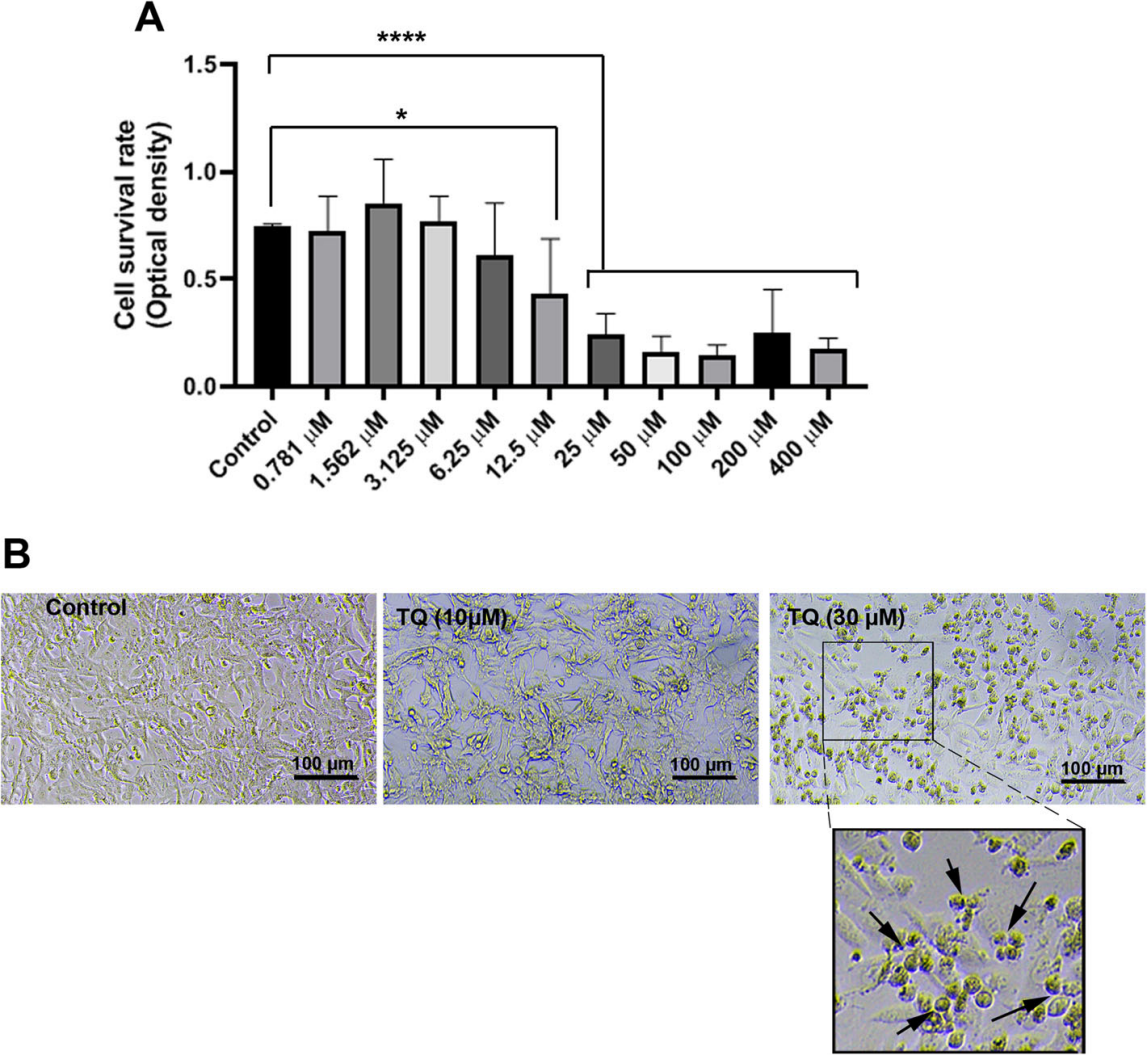
The viability of MDA-MB-231 triple-negative breast CSCs was evaluated using MTT assay after being exposed to various concentrations of TQ (**a**). Morphological changes were evaluated in CSCs. Cells lost their elongated shape and acquired round-form after being-treated with 10 and 30 μM TQ (**b**). One-Way ANOVA with Tukey posthoc analysis. *p < 0.05, and ****p < 0.0001

Cell survival and proliferation are critical events in the dynamic growth of cancer cells. In this line, an MTT assay was performed to assess the possible cytotoxic effect of TQ after 48 h in vitro. Based on data obtained from the MTT assay, we found that TQ could change the viability of MDA-MB-231 cells in a dose-dependent manner (Fig. 1a). According to our data, the inhibitory effect of TQ was started from 12.5 ϻM TQ compared to the control cells (*p* < 0.05; Fig. 1a). Treatment of CSCs with higher doses (25–400 ϻM TQ) contributed to prominent slop in the viability of cells compared to the control CSCs (*p* < 0.0001; Fig. 1a). The results from the MTT panel were the following previously published data [27–29]. In this regard, we selected doses 10 and 30 ϻM TQ for subsequent analyses. Bright-field imaging of cells exposed to 10 and 30 ϻM TQ revealed the morphological changes in MDA-MB-231 cells after 48 h. Based on our evaluation, the control cells exhibited a spindle-like appearance while the exposure of these cells to TQ altered morphological properties indicated by cell detachment and appearance of round-form cells (Fig. 1b). These features were abundant cells compared to the group 10 ϻM TQ.

### TQ impaired tubulogenesis capacity of MDA-MB-231 cells in vitro

**Fig. 2:**
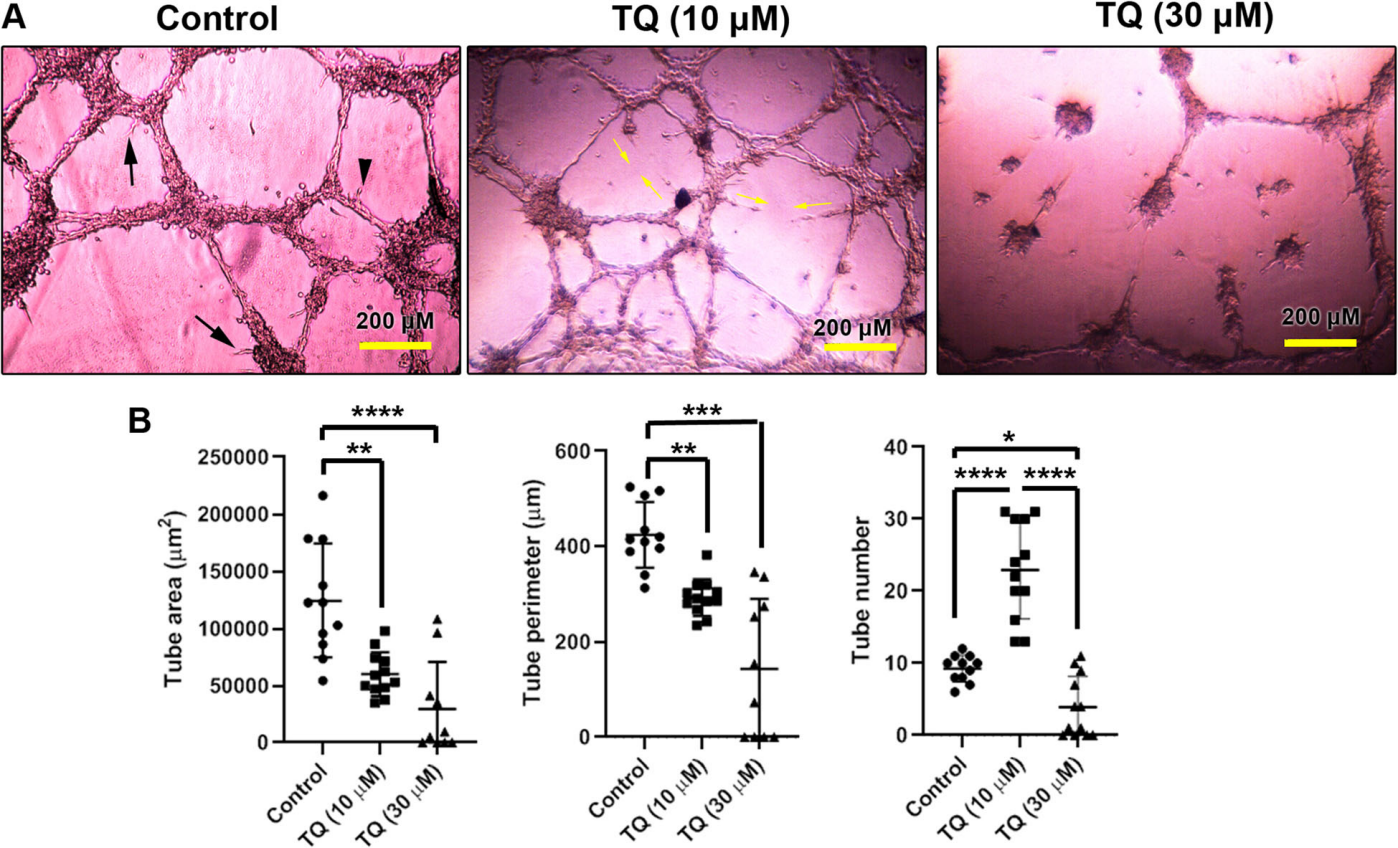
Measuring tubulogenesis capacity of CSCs on Matrigel substrate (**a**-**b**). The inhibitory effect of 10, 30 ϻM TQ was studied on MDA-MB-321 CSCs. Bright-field images showed the inhibition of tubulogenesis activity in CSCs after being-exposed to TQ. Black arrows: cell budding; small yellow arrows: anastomosis. One-Way ANOVA with Tukey posthoc analysis. *p < 0.05, **p < 0.01; ***p < 0.001 and ****p < 0.0001

To evaluate the impact of TQ on tubulogenesis, MDA-MB-231 cells were pre-incubated with two concentrations of TQ (10, 30 μM) for 24 h before seeding on Matrigel (Fig. 2a). Bright-field imaging revealed that these cells can be aligned to form tubes on the Matrigel substrate. We noted numerous budding and arborization and anastomosis (connection between tubular structures) control cells (black arrows and arrows head). The thickness of tubular walls seems larger compared to groups received 10 and 30 μM TQ, showing the potency of TQ to reduce cell alignment in the vascular wall (Fig. 2a). According to our data, the treatment of MDA-MB-231 cells with 10 and 30 μM TQ led to significant inhibition of tubulogenesis in which all values such as tube area, perimeter, and number were significantly inhibited compared to non-treated control cells (*p* < 0.05; Fig. 2b). Based on our data, TQ could inhibit the angiogenesis capacity of MDA-MB-231 cells in a dose-dependent manner. We found that the treatment of MDA-MB-231 cells with 30 μM TQ completely hampered the cell alignment and tube area compared to 10 μM TQ. In group 10 μM TQ, cells tended to form isolated micro-aggregates indicating the loss of appropriate attachment to beneath substrate. Despite the reduction of angiogenesis values in the 10 μM TQ group, cells with the capacity to maintain anastomosis were detectable in Matrigel substrate (yellow arrows). These features show that the incubation of MDA-MB-231 cells with 10 μM TQ could not abort the tubulogenesis capacity. Taken together, these data demonstrate that 48-h incubation of MDA-MB-231 cells with TQ could diminish the tubulogenesis capacity, leading to reduced angiogenesis.

### TQ altered Rhodamine 123 efflux and stemness capacity of MDA-MB-231 cells

**Fig. 3:**
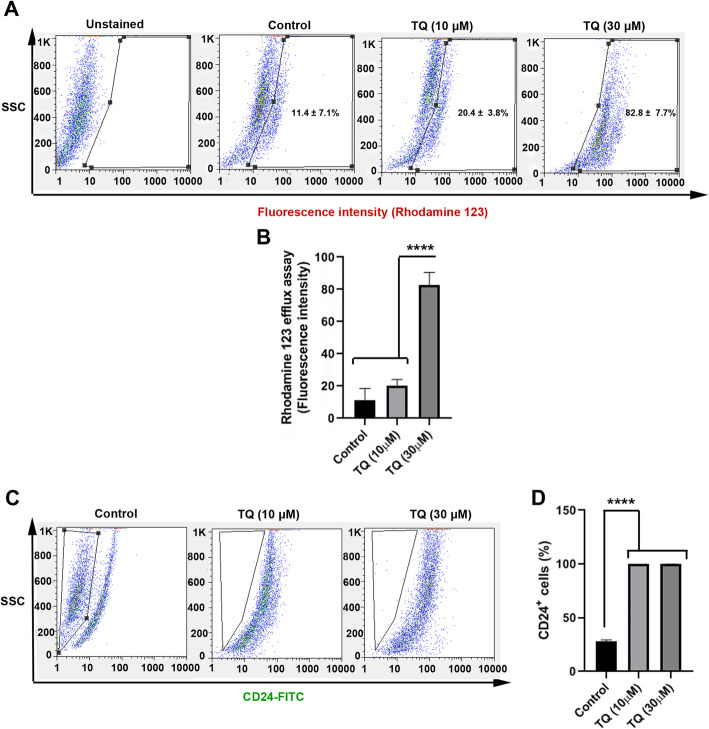
Flow cytometric analysis of Rhodamine 123 efflux capacity in CSCs after treatment with TQ (**a**). Treatment with TQ inhibited the Rhodamine 123 efflux capacity and increased accumulated intracellular Rhodamine 123 inside the CSCs (**a**-**b**). The percent of CD24^−^ CSCs was also determined using flow cytometry (**c**). Results showed the increase of CD24^+^ CSCs and stemness loss in condition containing TQ. One-Way ANOVA with Tukey posthoc analysis. ****p < 0.0001

The ability of Rhodamine efflux correlates with the ABC transporter function and osmotic pressure [30]. To this end, we performed flow cytometry analysis to measure the function of ABC transports in MDA-MB-231 cells after being-treated with TQ. Data showed that a small fraction of control cells (11.4 ± 7.1%) are Rhodamine 123-positive after 48 h while these values increased in TQ-treated groups (Fig. 3a). We observed 30 ϻM TQ significantly increased the percent of Rhodamine positive cells (82.8 ± 7.7%; *p* < 0.0001; Fig. 3b), indicating Rhodamine accumulation inside the cells. Despite the increase of Rhodamine 123 positive cells in 10 ϻM TQ, the differences did not reach statistically significant levels (20.4 ± 3.8% vs 11.4 ± 7.1%), showing appropriate tolerability of MDA-MB-231 cells to the low concentration of TQ. These data highlighted the potency of TQ to alter efflux capacity in MDA-MB-231 cells after 48 h which could diminish drug resistance capacity in human CSCs. To examine the possible effect of TQ on CSCs stemness, we also performed flow cytometric analysis of cells based on CD24 (Fig. 3c). In this study, we found that 48-h incubation of CSCs with TQ decreased stemness capacity by reducing the percent of CD24^+^ cells compared to the control group (*p* < 0.0001; Fig. 3d). Collectively, one could hypothesize that the treatment of MDA-MB-231 cells with TQ could decrease CSC resistance and stemness by modulating the function of the ABC transporter and protein levels of CD24.

### The combined effect of TQ plus PI3K and Wnt3a inhibitors on MDA-MB-231 cells

**Fig. 4:**
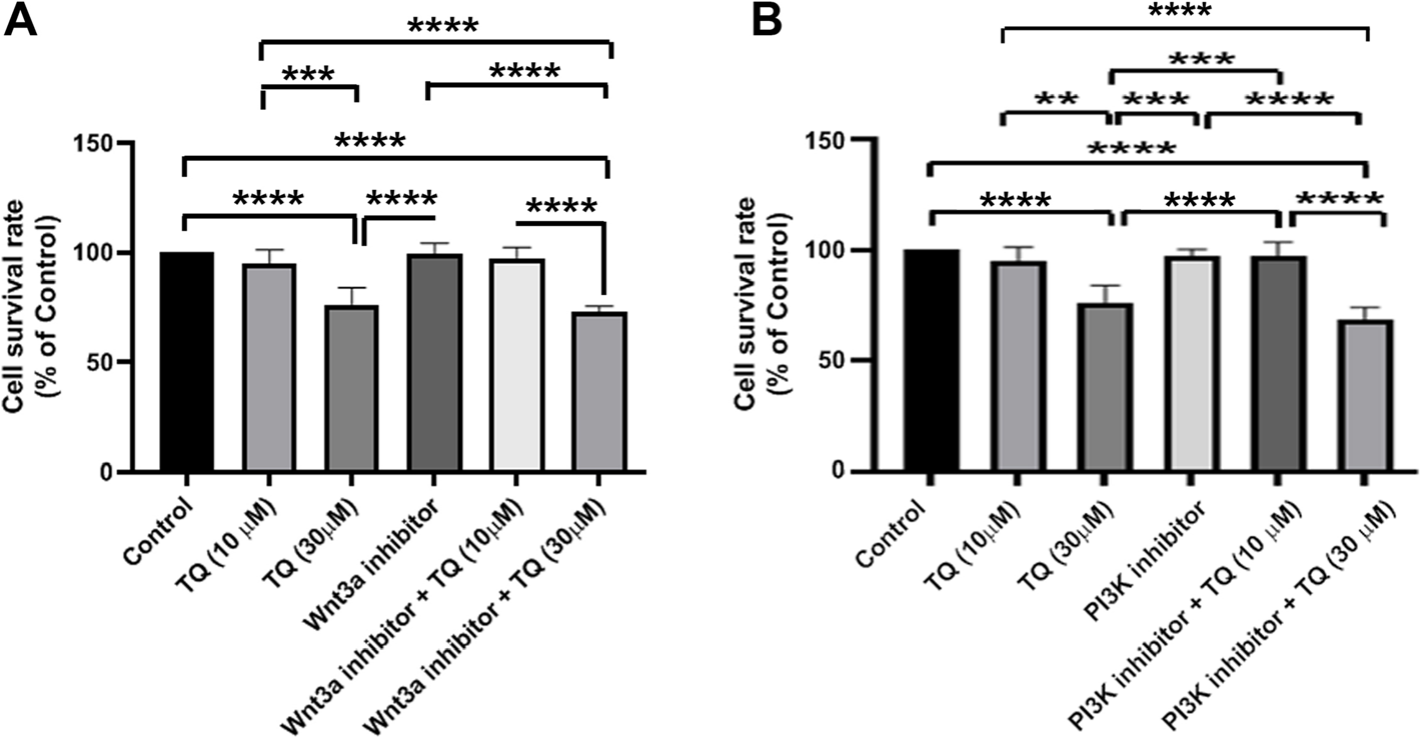
The combined effects of TQ, PI3K, and Wnt3a were evaluated on the viability of the MDA-MB-231 CSCs using MTT (**a**-**b**). Data showed that the combination of PI3K and Wnt3a inhibitors with TQ at a higher dose (30 μM) could significantly diminish CSCs viability as compared to the control group. One-Way ANOVA with Tukey posthoc analysis. **p < 0.01; ***p < 0.001 and ****p < 0.0001

In this panel, the combined effects of TQ, PI3K, and Wnt3a inhibitors were studied on the viability of the MDA-MB-231 cells after 48 h. Data showed that TQ could reduce CSCs survival rate in a dose-dependent manner in which 30 μM TQ treated cells showed reduced viability compared to the control cells (*p* < 0.0001; Fig. 4a, b). We noted that neither PI3K inhibitor nor Wnt3a inhibitor could diminish the viability of CSCs after 24 h. These data show that inhibition of PI3K and Wnt3a are not correlated with the survival rate in human MDA-MB-231 CSCs. The combination of PI3K inhibitors with TQ at a higher dose (30 μM) could significantly diminish cell viability as compared to the control group (Fig. 4a). A similar pattern was found in groups treated with Wnt3a inhibitor and 30 μM TQ (Fig. 4a). These data show that TQ could decrease the survival rate of CSCs in a dose-dependent manner. Additionally, the reduction of survival rate in groups treated with inhibitors + TQ could be related to the inhibitory effect of TQ rather than inhibitors’ activity.

### TQ blunted the stimulatory effect of VEGF, EGF, and FGF on MDA-MB-231 cells

**Fig. 5:**
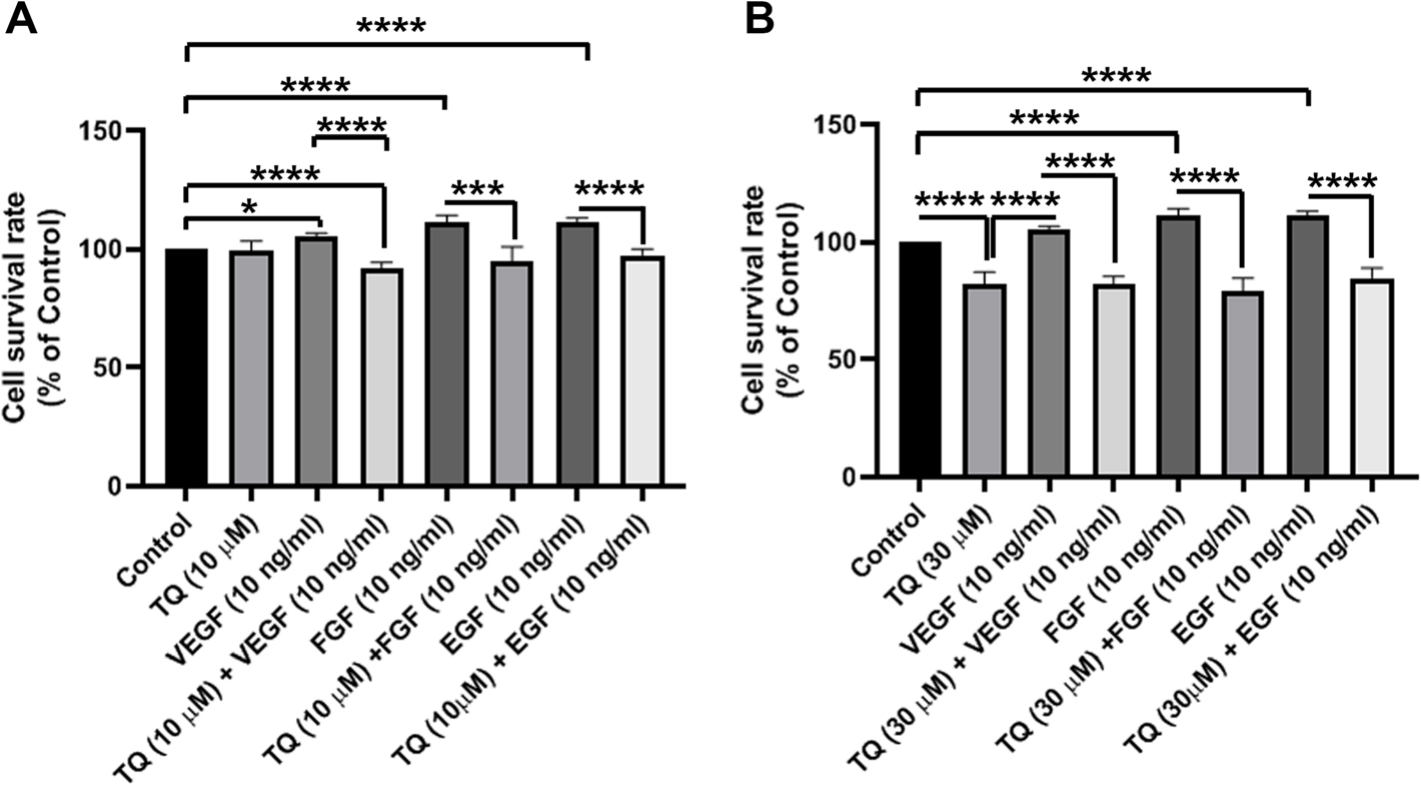
The stimulatory effect of growth factors such as VEGF, EGF, and FGF was evaluated on the viability of MDA-MB-231 CSCs pre-treated with TQ using MTT assay (**a**-**b**). Data showed that pre-treatment of MDA-MB-231 CSCs with TQ inhibited the stimulatory effects of all growth factors compared to matched control groups. One-Way ANOVA with Tukey posthoc analysis. *p < 0.05, **p < 0.01; ***p < 0.001 and ****p < 0.0001

The potency of different factors such as VEGF, FGF, and EGF was evaluated on the viability of MDA-MB-231 cells pre-treated with TQ after 48 h. Data revealed an increased cell survival rate in all groups treated with 10 ng/ml of VEGF, EGF, and FGF compared to the control group (*p* < 0.05; Fig. 5a-b). According to our data, pre-treatment of MDA-MB-231 cells with TQ inhibited the stimulatory effects of all growth factors compared to matched control groups (*p* < 0.05; Fig. 5a-b). These data showed TQ has the potential to desensitize CSCs to respond to angiogenic factors, VEGF, and proliferative agents such as EGF and FGF in vitro, showing prophylactic potency of TQ to inhibit different bioactivities of MDA-MB-231 cells. To investigate the possible effect of TQ on MDA-MB-231 cells clonogenic capacity, we performed a spheroid formation assay. Based on our observation, these cells could not form microaggregates and spheroids (data not shown).

### Combination of TQ with chemical inhibitors suppressed migration capacity and MendT

**Fig. 6:**
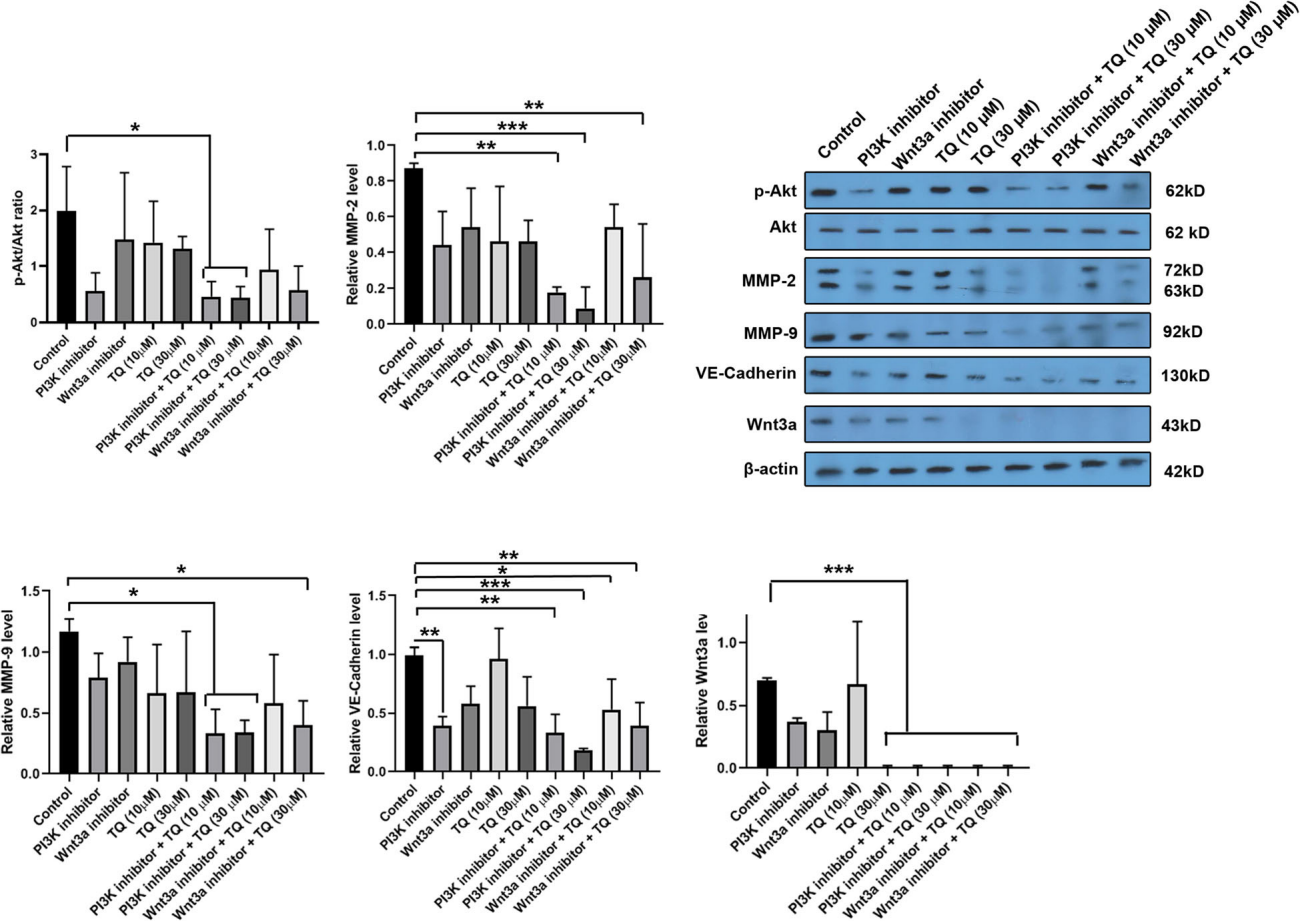
Western blotting. The combined effects of TQ and PI3K, and Wnt3a inhibitors on the migration and angiogenic differentiation of the MDA-MB-231 CSCs. Western blot results showed that TQ could decrease the pAkt/Akt ratio and suppress the protein level of, MMP-2, MMP-9, VE-cadherin, Wnt3a in a dose-dependent manner. One-Way ANOVA with Tukey posthoc analysis. *p < 0.05, **p < 0.01; ***p < 0.001

To investigate the effect of TQ on MDA-MB-231 cell migration and angiogenic differentiation, we performed western blotting (Fig. 6). The effectors such as PI3K and Wnt3a participate in different activities of cancer cells [31, 32]. To this end, specific inhibitors of PI3K (Ly294002, 10 μM) and Wnt3a (LGK974, 10 μM) were used to treat MDA-MB-231 cells in the presence of TQ. Data indicated that TQ (10, 30 μM) plus PI3K inhibitor decreased significantly the pAkt/Akt ratio compared to the control group (*p* < 0.05; Fig. 6) while the combination of Wnt3a inhibitor with TQ did not alter the pAkt/Akt ratio. Monitoring the levels of MMP-2 and -9 showed a slight decrease, but non-significant, reduction in groups received 10 and 30 μM TQ for 48 h. Interestingly, co-treatment of MDA-MB-231 cells with TQ and Wnt3a and PI3K inhibitors suppresses the protein-coding of MMP-2 and -9. In our study, 30 μM TQ had superiority to inhibit the synthesis of MMP-2 and -9 compared to the groups 10 μM TQ. The vasculogenic potential of MDA-MB-231 cells was also assessed by monitoring endothelial cells specific markers, namely VE-cadherin, after being-treated with TQ for 48 h. Data demonstrated that the incubation of CSCs with PI3K inhibitor, but not Wnt3a inhibitors, hampered MendT of MDA-MB-231 cells by suppressing VE-Cadherin levels compared to non-treated control cells. Although both concentrations of TQ (10 and 30 μM) decreased the levels of VE-cadherin the values did not reach statistically significant compared to the control cells. The combination of two types of inhibitors Ly294002 and LGK974 with TQ could significantly abort MendT capacity in human breast CSCs after 48 h as compared to the control group. We also noted that the exposure of MDA-MB-231 CSCs with TQ plus Wnt3a inhibitor could reduce protein levels of Wnt3a compared to the control cells. Interestingly, the Wnt3a inhibitor could not significantly diminish the Wnt3a level alone (Fig. 6). Taken together, these data show that TQ could be touted as complementary phyto-compound with tumoricidal activity to alter the protein levels of intracellular effectors such as PI3K and Wnt3a which are important in multiple cancer cells bioactivities such as metastasis and vasculogenic mimicry.

## Discussion

Among various types of anaplastic changes, breast cancer remained one of the highest death cases globally [1]. The promotion of pro-angiogenesis signaling as well as VM seems to be an efficient phenomenon in the development of tumor size, metastasis, and invasion to the remote sites [4, 5]. Of note, the emergence of CSCs in the tumor niche contributes to resistance against different chemotherapeutic agents such as doxorubicin, etc., leading to high-rate mortality [33–35]. A plethora of scientific literature confirmed the existence of different strategies driven by CSCs to increase the survival of normal cancer cells [15, 18]. For instance, it has been determined that the CSCs exhibit distinct angiogenic and vasculogenic properties inside the tumor niche by releasing multiple pro-angiogenesis factors and differentiation into endothelial lineage [5, 6]. In parallel with chemotherapeutic agents, natural products especially phyto-compounds are at the center of attention in the prevention and therapy of cancer diseases [5]. TQ is a bioactive component originated from *Nigella sativa* that has been extensively used in folk medicine against many diseases, including cancers [19]. Here, we investigated the tumoricidal effects of TQ on human MDA-MB-231 CSCs, CD24^−^/CD44^+^, after 48 h.

We found that TQ could decrease the viability of MDA-MB-231 cells in a dose-dependent manner after 24 h. Consistent with the current data, Peng and co-workers previously found an increased apoptotic change and the suppression of the NF-ƙB signaling pathway in the human Osteosarcoma SaOS-2 cell line after exposure to the TQ [19]. Other experiments showed the accumulation of reactive oxygen species, suppression of NF-ƙB, STAT-3 inhibition participate in the suppression of cancer cells in the presence of TQ [27, 36]. These data showed that TQ could decrease the survival rate of normal cancer cells and CSCs, however, further experiments are needed to discover the mechanism of action of TQ in CSCs.

To the best of our knowledge, the tumoricidal effects of TQ have been investigated in multiple cancer types but there are few experiments correlated with the angiogenesis switch on/off in the CSCs after being-treated with TQ. Commensurate with these comments, the primary aim of this study was to study the anti-angiogenesis capacity of TQ on human CSCs. We hypothesize that the inhibition of CSCs angiogenesis and vasculogenesis capacity will help us to slow down the expansion and progression of tumor cells to the other sites. Here, we showed that 48-h incubation of MDA-MB-231 cells with TQ suppressed the tubulogenesis capacity of CSCs on the Matrigel substrate compared to the non-treated control cells. We found the superiority of 30 μM TQ in the inhibition of tubulogenesis as compared to the 10 μM TQ, showing a dose-dependent activity of TQ in the inhibition of angiogenesis. 30 μM TQ had the potential to disrupt the cell-to-cell connection and alignment on the Matrigel surface, leading to the formation of numerous localized micro-aggregates compared to the control and 10 μM TQ, the increase of TQ concentration to 30 μM diminished the number of budding and arborization. In support of our data, the anti-angiogenic effects of TQ have been reported in in vivo zebrafish angiogenesis model and in a xenograft human prostate cancer [21, 29]. Our results indicated that TQ suppressed VE-cadherin levels compared to non-treated control cells.

We also monitored the survival of CSCs in the presence of different angiogenesis growth factors such as FGF, VEGF, and EGF. These factors are pro-angiogenic agents that stimulated cancer cell proliferation and neo-angiogenesis inside tumor stroma [37]. Our data showed the increase of CSCs survival after being-treated with these factors. Pre-treatment with 30 μM TQ inhibited the stimulatory effect of FGF, VEGF, and EGF in MDA-MB-231 cells, showing the lack of cellular response to pro-angiogenesis factors.

The potency of CSCs to exclude different therapeutic agents seems to be correlated to the multipotentiality state [35]. In this regard, it has been shown that the ATP Binding Caste protein (ABC) transporters are highly expressed in these cells which can able these cells to resist again different insulting conditions [38]. To address these issues, we performed a Rhodamine 123 exclusion assay. Data showed that the incubation of CSCs with TQ could blunt the exclusion of Rhodamine 123 compared to the control CSCs. These features coincided with the stemness feature removal and increase of surface membrane-bounded CD24 expression. Previous investigations have revealed a close relationship between the stemness feature (the cellular distribution of CD24, CD44, and CD133) with efflux capacity [34, 39]. Therefore, one could hypothesize that incubation with TQ could sensitize the CSCs via the accumulation of different chemotherapeutic agents.

To understand the participation of signaling pathways inside CSCs to modulate VM after being treated with TQ, we measured the protein levels of PI3K and Wnt3a. Several studies reported that TQ suppressed tumor progression via PI3K/Akt and Wnt3a pathways in various types of cancer [40–43]. We found that the incubation of CSCs with PI3K and Wnt3a inhibitors and TQ could suppress the protein levels of MMP-2 and -9, showing an inhibitory effect of TQ on CSCs migration and metastasis. The incubation of CSCs with inhibitors and TQ alone did not yield statistically significant results compared to the control cells. Therefore, it seems that the combination of TQ with other chemical agents could be a strategy to inhibit the metastasis of CSCs. The blockade of VEGFR2/PI3K/Akt has been shown to suppress the angiogenesis capacity of cancer cells [44]. Here, we also highlighted the decrease of p-Akt/Akt ratio in CSCs after treatment with TQ plus PI3K or Wnt3a inhibitors [41, 42]. Previously, researchers have proved that TQ inhibited tumor growth and angiogenesis through Akt and ERK signaling pathways [29]. It seems that both Wnt3a and PI3K/Akt are two possible pathways involved in CSCs survival and angiogenesis/VM. The potency of MendT capacity was also monitored in MDA-MB-231 cells after being-treated with TQ. Based on our data, TQ in combination with PI3K and Wnt3a inhibitors decreased the protein levels of VE-cadherin, showing the inhibition of CSCs differentiation toward endothelial lineage. The major mechanism of TQ anti-angiogenesis effects is done via the MendT inhibition by PI3K and Wnt3a signaling pathway. Similar to data from the in vitro tube formation assay, western blotting confirmed the inhibition of VE-cadherin in the group that received TQ. VE-cadherin could promote VM network formation through the connection of ECs. VE-cadherin is highly expressed in ECs to stabilize the EC-to-EC connection [45]. Overall, TQ decreased the angiogenic potential of CSCs via the production of VE-cadherin and stemness removal. Interestingly, the critical association of VE-cadherin was determined with erythropoietin-producing hepatocellular carcinoma-A2 which in turn could engage the PI3K/Akt signaling pathway [46]. It seems that the inhibition of the PI3K/Akt signaling pathway contributes to pleiotropic effects. It has been shown that the inhibition of this axis could decrease the activity MMP-2, − 14, and the Ln-5γ2 chain’s cleavage [47]. There are several limitations to our study. It seems that different molecular machinery and signaling pathways are involved in the VM potency of CSCs. Whether these pathways are influenced by TQ is the subject of interest. The efficiency of TQ supplementation along with other chemotherapeutic agents should be monitored in in vivo models.

### Conclusions

**Fig. 7:**
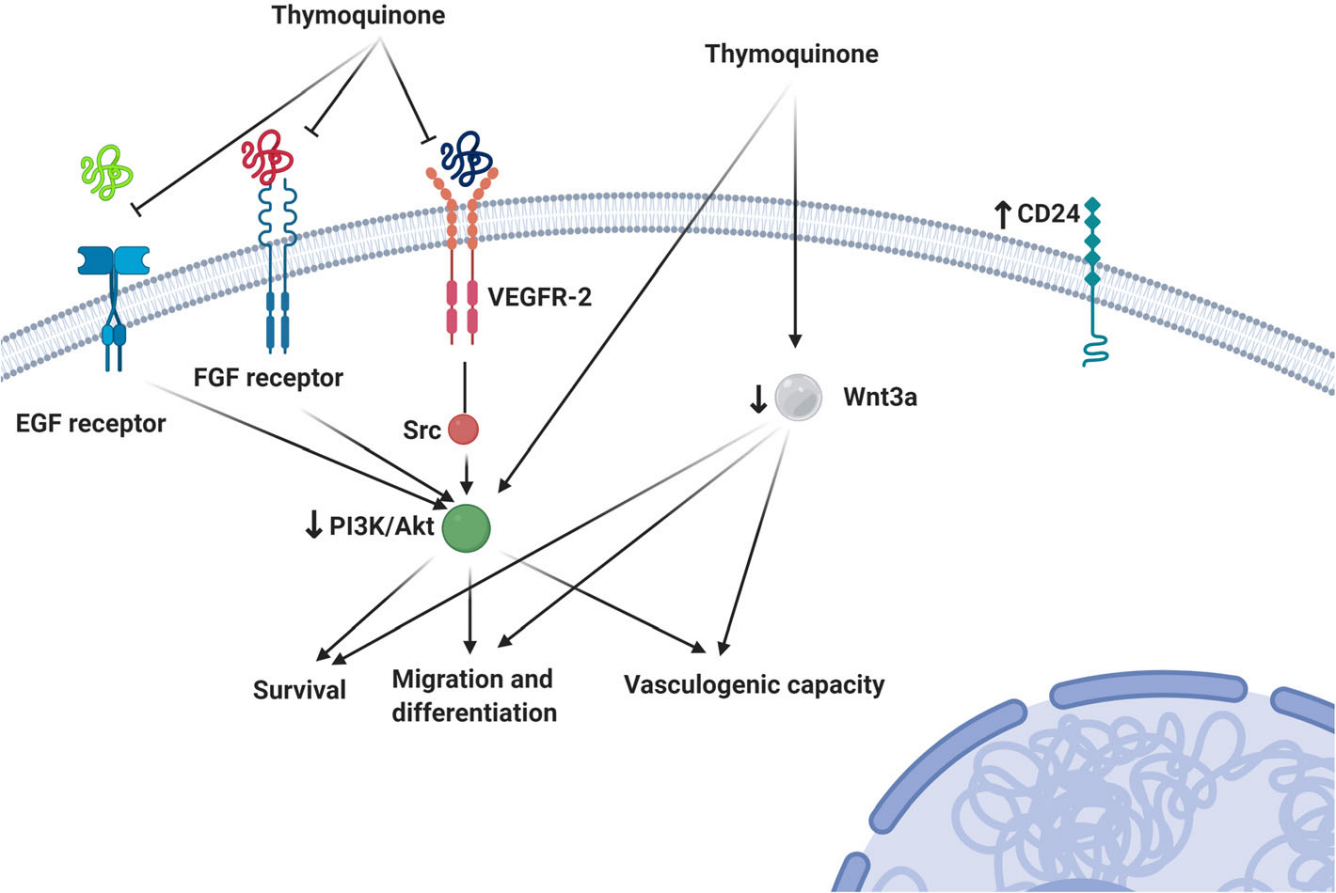
The possible effect of TQ on human breast cancer via the modulation of different signaling pathways

Taken together, our results indicated that TQ suppressed VM network formation and metastasis in breast cancer cell line MDA-MB-231 through modulating PI3K, Wnt3a, VE-cadherin pathways which are essential signaling in VM network formation (Fig. 7). Thus TQ together with anti-angiogenic therapies may be a novel therapeutic agent in the elimination of VM in breast cancer.

## Data Availability

All data generated or analyzed during are included in this published article.

## Abbreviations

FGF: Basic fibroblast growth factor
CSCs: Cancer stem cells
ECs: Endothelial cells
EGF: Epidermal growth factor
FBS: Fetal Bovine Serum
MMP: Matrix metalloproteinase
MendT: Mesenchymal-to-endothelial transition
MTT: 3-(4, 5-Dimethylthiazol-2-yl)-2, 5-Diphenyltetrazolium Bromide
PBS: Phosphate-buffered saline
TQ: Thymoquinone
VEGF: Vascular endothelial growth factor
VM: Vasculogenic mimicry
SDS: sodium dodecyl sulfate
