# Correction to: Thymoquinone inhibited vasculogenic capacity and promoted mesenchymal-epithelial transition of human breast cancer stem cells

**DOI:** 10.1186/s12906-021-03414-y

**Published:** 2021-10-25

**Authors:** Sanya Haiaty, Mohammad-Reza Rashidi, Maryam Akbarzadeh, Ahad Bazmani, Mostafa Mostafazadeh, Saba Nikanfar, Zohre Zibaei, Reza Rahbarghazi, Mohammad Nouri

**Affiliations:** 1 Student Research Committee, Tabriz University of Medical Sciences, Tabriz, Iran; 2 Department of Biochemistry and Clinical Laboratories, Tabriz University of Medical Sciences, Tabriz, Iran; 3 Stem Cell and Regenerative Medicine Institute, Tabriz University of Medical Sciences, Tabriz, Iran; 4 Department of Biochemistry, Erasmus University Medical Center, Rotterdam, the Netherlands; 5 Department of Pathobiology, Faculty of Veterinary Medicine, Ferdowsi University Of Mashhad, Mashhad, Iran; 6 Infectious and Tropical Diseases Research Center, Tabriz University of Medical Sciences, Tabriz, Iran; 7 Stem Cell Research Center, Tabriz University of Medical Sciences, Imam Reza St., Golgasht St, Tabriz, Iran; 8 Departmnt of Applied Cell Sciences, Faculty of Advanced Medical Sciences, Tabriz University of Medical Sciences, Tabriz, Iran

**Correction to: BMC Complement Med Ther 21, 83 (2021)**

10.1186/s12906-021-03246-w

Following publication of the original article [1], the authors identified an error in the affiliations ordering, the author name of Ahad Bazmani, and the affiliations of research center in the Acknowledgments section. Corrections are below:

Sanya Haiaty^1,2^, Mohammad-Reza Rashidi^3^, Maryam Akbarzadeh^2,4^, Ahad Bazmani^5,6^, Mostafa Mostafazadeh^1,2^, Saba Nikanfar^1,2^, Zohre Zibaei^1,6^, Reza Rahbarghazi^3,7,8*†^ and Mohammad Nouri^3,7*†^

^1^Student Research Committee, Tabriz University of Medical Sciences, Tabriz, Iran.

^2^Department of Biochemistry and Clinical Laboratories, Tabriz University of Medical Sciences, Tabriz, Iran.

^3^Stem Cell and Regenerative Medicine Institute, Tabriz University of Medical Sciences, Tabriz, Iran.

^4^Department of Biochemistry, Erasmus University Medical Center, Rotterdam, the Netherlands.

^5^Department of Pathobiology, Faculty of Veterinary Medicine, Ferdowsi University Of Mashhad, Mashhad, Iran.

^6^Infectious and Tropical Diseases Research Center, Tabriz University of Medical Sciences, Tabriz, Iran.

^7^Stem Cell Research Center, Tabriz University of Medical Sciences, Imam Reza St., Golgasht St, Tabriz, Iran.

^8^Departmnt of Applied Cell Sciences, Faculty of Advanced Medical Sciences, Tabriz University of Medical Sciences, Tabriz, Iran.

The incorrect author name is: Ahad Bazmany

The correct author name is: Ahad Bazmani

**Acknowledgments**

We special thanks to the Infectious and Tropical Diseases Research Center, Tabriz University of Medical Sciences for their assistance in the progression of my thesis.

The original article [1] has been updated.
